# Chrysanthemum WRKY gene *DgWRKY5* enhances tolerance to salt stress in transgenic chrysanthemum

**DOI:** 10.1038/s41598-017-05170-x

**Published:** 2017-07-06

**Authors:** Qian-yu Liang, Yin-huan Wu, Ke Wang, Zhen-yu Bai, Qing-lin Liu, Yuan-zhi Pan, Lei Zhang, Bei-bei Jiang

**Affiliations:** Department of Ornamental Horticulture, Sichuan Agricultural University, 211 Huimin Road, Wenjiang District, Chengdu, Sichuan 611130 P. R. China

## Abstract

WRKY transcription factors play important roles in plant growth development, resistance and substance metabolism regulation. However, the exact function of the response to salt stress in plants with specific WRKY transcription factors remains unclear. In this research, we isolated a new WRKY transcription factor *DgWRKY5* from chrysanthemum. *DgWRKY5* contains two WRKY domains of WKKYGQK and two C_2_H_2_ zinc fingers. The expression of *DgWRKY5* in chrysanthemum was up-regulated under various treatments. Meanwhile, we observed higher expression levels in the leaves contrasted with other tissues. Under salt stress, the activities of superoxide dismutase (SOD), peroxidase (POD) and catalase (CAT) enzymes in transgenic chrysanthemum were significantly higher than those in WT, whereas the accumulation of H_2_O_2_, O_2_^−^ and malondialdehyde (MDA) was reduced in transgenic chrysanthemum. Several parameters including root length, root length, fresh weight, chlorophyll content and leaf gas exchange parameters in transgenic chrysanthemum were much better compared with WT under salt stress. Moreover, the expression of stress-related genes *DgAPX*, *DgCAT*, *DgNCED3A*, *DgNCED3B*, *DgCuZnSOD*, *DgP5CS*, *DgCSD1* and *DgCSD2* was up-regulated in *DgWRKY5* transgenic chrysanthemum compared with that in WT. These results suggested that *DgWRKY5* could function as a positive regulator of salt stress in chrysanthemum.

## Introduction

Environmental stress such as cold, high salinity, metal ions, drought and other abiotic stress will severely inhibit plant growth progress, cause morphological and physiological changes, and even death. In plant stress resistance system, transcription factors regulate the expression of functional genes, which are essential for plant stress response^1^. Many transcription factors related to plant stress tolerance have been identified, such as DREB, bZIP, NAC, WRKY^2, 3^. These transcription factors combine with specific cis-acting elements to constitute a regulatory network, specifically regulate the expression of various stress-related genes, and improve the adaptability of plants to environmental stress.

WRKY transcription factors are part of the largest families of transcription factors in plants. The WRKY family was named based on the WRKY domain, which been composed of a highly conserved sequence WRKYGQK at its N-terminus with a C_2_H_2_- or C_2_HC-type zinc-finger motif^4^. And WRKY protein was divided into three groups (I, II, and III) and various subgroups were based on their original structure^5^. WRKY group I proteins contain two WRKY domains and two C_2_H_2_ zinc-fingers. Other studies have shown that WRKY transcription factors can specifically bind to the W box [TTGAC(C/T)], and interact with the target gene promoters^6^. In addition to involve in plant growth development and metabolic regulation, WRKY proteins are also resistant to stress and senescence^7^. *Arabidopsis thaliana WRKY25*, *WRKY26*, and *WRKY33* positively regulated heat stress-response, and these three proteins interacted in function and played overlapping and synergetic roles in plant heat tolerance^8^. *OsWRKY30* functioned as a positive regulator in rice resistance of disease via a SA signaling pathway^9^. Overexpression of *TaWRKY2* in transgenic *Arabidopsis* plants enhanced tolerance to drought and salt stresses, and overexpression of wheat WRKY gene *TaWRKY19* increased the salt, drought, and freezing tolerance in transgenic plants^10^. Moreover, overexpression of chrysanthemum genes *DgWRKY1* and *DgWRKY3* enhanced tolerance to salt stress in tobacco^11, 12^.

Chrysanthemum is a widely grown ornamental plant around the world, which is sensitive to salinity^13^. High salinity can cause chrysanthemum leaf chlorosis, inhibit plant growth, and sometimes even kill plants. In chrysanthemum salt tolerance experiment, we isolated and identified a novel group I WRKY transcription factor, *DgWRKY5*, which was demonstrated to be induced by salinity. And we found that overexpression of *DgWRKY5* in chrysanthemum plants increased salt stress tolerance by regulating physiological changes and downstream genes.

## Results

### Isolation and characterization of *DgWRKY5*

*DgWRKY5* contained a complete ORF (open reading frame) of 1603bp encoding a protein of 540 amino acids with the theoretical molecular weight of 60.0KDa (Fig. S1). Multiple alignments between *DgWRKY5* and the other homologous proteins via DNAMAN showed that *DgWRKY5* contains two WRKY domains of WKKYGQK and two C_2_H_2_ zinc-fingers (Fig. S2). Therefore, we divided *DgWRKY5* into the WRKY family group I based on the conserved domain features. The phylogenetic analysis showed that *DgWRKY5* is more closely linked to *AtWRKY26* from *Arabidopsis thaliana* (Fig. S3).

### Expression analysis of *DgWRKY5* under various stresses

**Figure 1:**
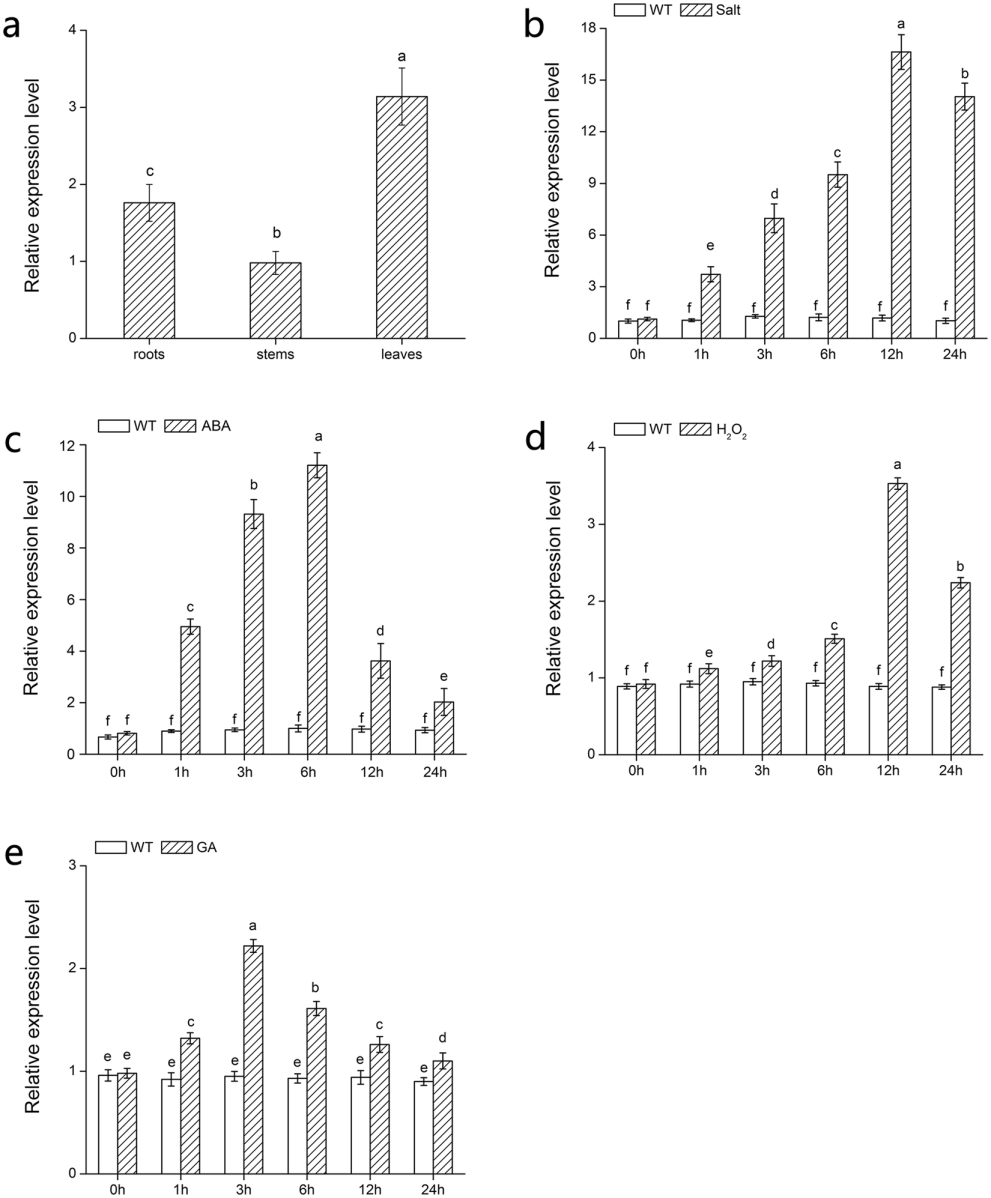
Analysis of *DgWRKY5* expression in different tissues and in response to various treatments. (**a**) Expression patterns of *DgWRKY5* in roots, stems and leaves. (**b**) Salt. (**c**) ABA. (**d**) H_2_O_2_. (**e**) GA. Data represent means and standard errors of three replicates. Different letters above the columns indicate significant differences (P < 0.05) on the basis of Duncan’s multiple range test.

Expression profiles of *DgWRKY5* in different tissues of chrysanthemum were examined using qRT-PCR. The result showed that higher relative expression of *DgWRKY5* was observed in leaves and roots, while lower relative expression was observed in stems (Fig. 1a). Expression patterns of *DgWRKY5* gene in leaves under salt stress were also analyzed through qRT-PCR. For salt stress, the expression level of *DgWRKY5* increased gradually after 1 hour, and reached the peak at 12 hours, thereafter *DgWRKY5* expression levels decreased, but remained at a higher level opposed to untreated control (Fig. 1b). Under ABA treatment, transcript level reached a peak at 6 hours and then declined (Fig. 1c). In response to H_2_O_2_, *DgWRKY5* expression was induced after 12 hours of treatment and then subsequently dropped (Fig. 1d). During GA treatment, *DgWRKY5* transcript level accumulated, reaching its maximum at 3 hours and gradually diminishing (Fig. 1e). These results suggest that *DgWRKY5* expression was induced under various stresses.

### Salt tolerance analysis of *DgWRKY5* in transgenic chrysanthemum

**Figure 2:**
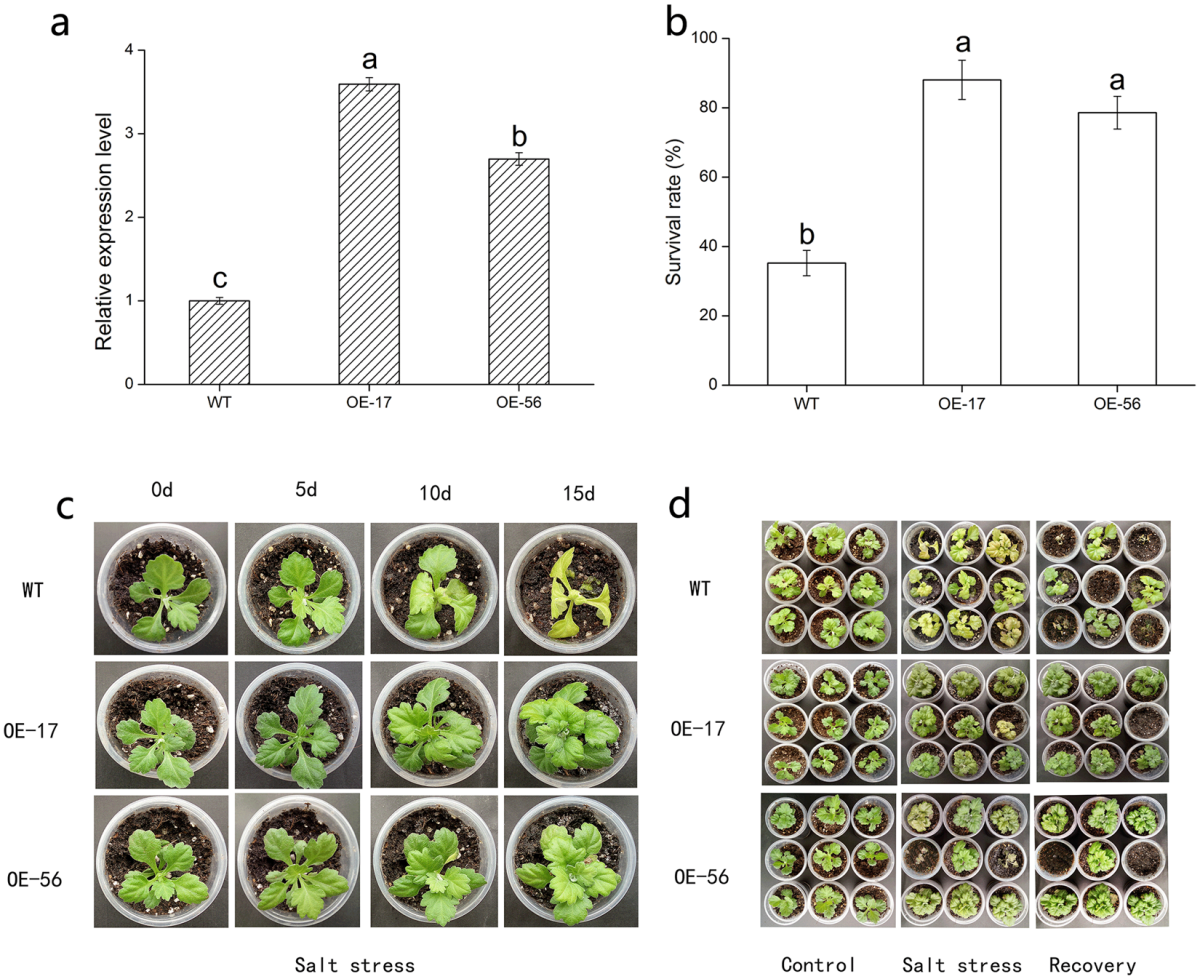
Salinity tolerance of transgenic chrysanthemum plants overexpressing *DgWRKY5* (OE-17 and OE-56). (**a**) The expression level of *DgWRKY5* in wild type (WT) and transgenic lines. (**b**) The survival rates of transgenic lines and WT after two weeks of recoveries. (**c**) Phenotypic comparison of *DgWRKY5* transgenic lines (OE-17 and OE-56) and WT under salt stress. (**d**) Transgenic lines and WT grown under non-stress and salt stress conditions, followed by a recovery.

To investigate whether overexpression of *DgWRKY5* enhances salt tolerance, transgenic chrysanthemum lines overexpressing *DgWRKY5* were generated, and *DgWRKY5* transcription levels in eight transgenic lines were detected by qRT-PCR (Fig. S4a–c). *DgWRKY5* transcription levels were substantially (*P < *0.05) higher in OE-17 and OE-56 than in WT (Fig. 2a). Therefore, we selected OE-17 and OE-56 lines plants for further salt tolerance evaluation. Under normal conditions, the phenotype of transgenic plants and WT plants had no significant difference. However, leaves of WT plants were evident wilting and yellowing compared with transgenic lines OE-17 and OE-56 under salt stress (Fig. 2c,d). Two weeks recovery after salinity, the survival rate in WT was 35%, while the survival rates in transgenic lines OE-17 and OE-56 were 88% and 78%, respectively, which were two times as many as that in WT (Fig. 2b).

### Accumulation of H_2_O_2_, O_2_^−^ and MDA in *DgWRKY5* transgenic chrysanthemum plants under salt stress

**Figure 3:**
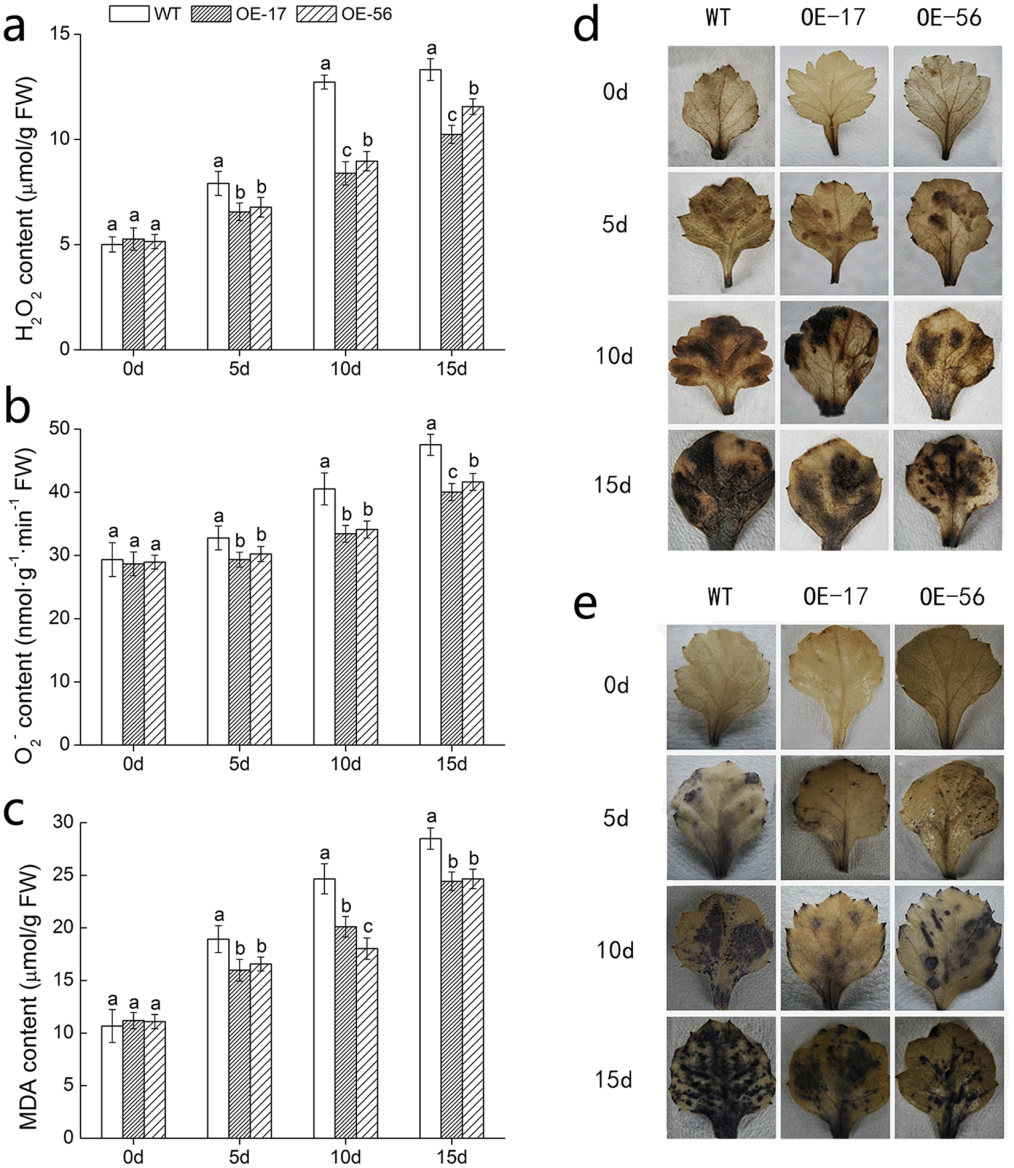
Analysis of H_2_O_2_, O_2_^−^ and MDA in *DgWRKY5* transgenic lines and WT under salt stress. (**a**) H_2_O_2_. (**b**) O_2_^−^. (**c**) MDA. (**d**) DAB staining. (**e**) NBT staining.

In order to study salt tolerance differences between *DgWRKY5* transgenic chrysanthemum (OE-17 and OE-56) and WT, H_2_O_2_, O_2_^−^ and MDA were determined. MDA is a product of membrane lipid peroxidation, which can directly reflect the degree of membrane damage. Under routine conditions, the content of H_2_O_2_, O_2_^−^ and MDA between wild-type and transgenic plants had no significant (*P < *0.05) difference (Fig. 3a–c). Under salt stress, the H_2_O_2_, O_2_^−^ and MDA content of all lines increased slightly after exposed to salinity. However, the accumulation of H_2_O_2_, O_2_^−^ and MDA in transgenic lines was much smaller than WT in response to salt stress (Fig. 3a–c). To intuitively understand the oxidation status of chrysanthemum, the accumulation of H_2_O_2_ and O_2_^−^, two major reactive oxygen species, was detected with 3, 3′-diaminovenzidine (DAB) staining and nitroblue tetazolium (NBT) staining. The consequence of research indicated that the accumulation of H_2_O_2_ and O_2_^-^ in leaves of transgenic lines was significantly (*P < *0.05) lower, compared with WT plants (Fig. 3d, e). These data suggested that WT plants suffered more severe membrane damage than *DgWRKY5* transgenic plants. And it indicated that overexpression of *DgWRKY5* conferred transgenic chrysanthemum greater tolerance to the oxidative stress associated with salt stress.

### Accumulation of osmotic regulators in *DgWRKY5* transformed chrysanthemum plants under salt stress

**Figure 4:**
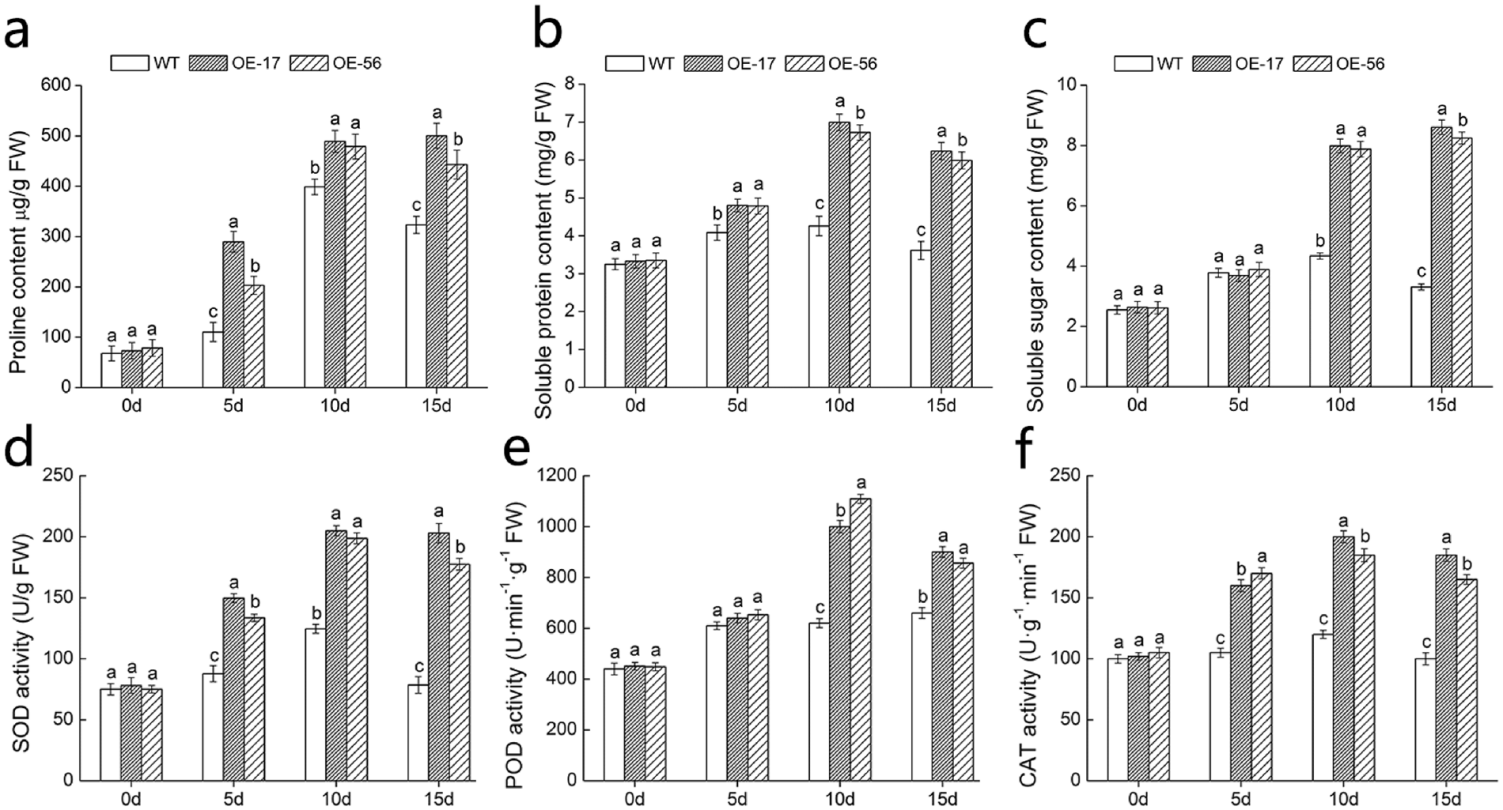
Analysis of physiological effects between WT and *DgWRKY5* transgenic chrysanthemum lines (OE-17 and OE-56) under non-stress conditions and salt stress conditions at various time points (0, 5, 10 and 15d). (**a**) Proline. (**b**) Soluble protein. (**c**) Soluble sugar. (**d**) SOD. (**e**) POD. (**f**) CAT.

In order to examine the effect of osmotic adjustment on the salt tolerance between WT and transgenic plants, we determined the contents of soluble protein, soluble sugar and proline in transgenic lines and wild-type plants (Fig. 4a–c). These three osmotic regulators had no significant (*P < *0.05) difference between transgenic lines and wild-type plants under non-stress conditions. With the increase of salinity, the proline content of wild type and transgenic lines increased rapidly, and the accumulation in transgenic lines was significantly (*P < *0.05) higher than in wild type (Fig. 4a). In addition, after 5 days of salt processing, the content of soluble protein and soluble sugar in wild-type and transgenic lines was changed rarely, then with the increase of salinity, the content of transgenic plants in the following 10 days increased by nearly twice as much as WT (Fig. 4a–c). These osmotic adjustment substances were positively correlated with the salt tolerance of plants. These results indicated that overexpression of *DgWRKY5* plants increased salt tolerance by enhancing osmotic regulators.

### Analysis of antioxidant enzyme activity in *DgWRKY5* transformed chrysanthemum plants under salt stress

Antioxidant enzymes are the vital part in resistance of plants to stress^14^. So we measured the activities of SOD, POD and CAT in both WT and transgenic lines. The activities of SOD, POD and CAT in WT and transgenic plants remained the same during normal condition (Fig. 4d–f). However, with the increase of salinity, the activities of SOD, POD and CAT in transgenic plants rapidly raised and remained high levels, which were markedly (*P < *0.05) higher than WT plants (Fig. 4d–f). These results suggested that salt tolerance in transgenic chrysanthemum was enhanced by the boost of antioxidant enzyme activities.

### Effect of salt stress on growth, chlorophyll content and photosynthesis in *DgWRKY5* transformed chrysanthemum plants

**Figure 5:**
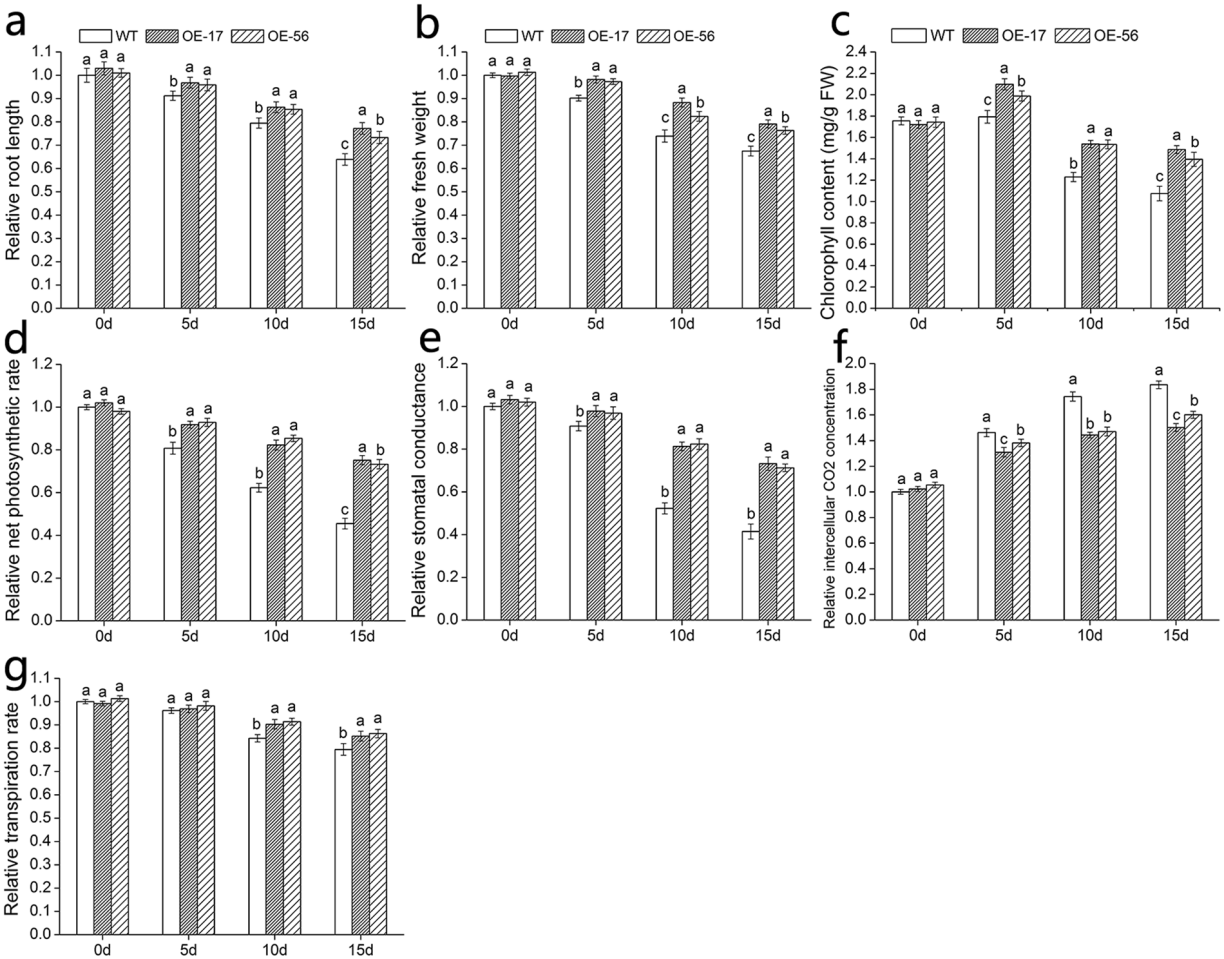
Assay of root length, fresh weight, chlorophyll content and leaf gas exchange parameters in *DgWRKY5* transgenic lines and WT under salt stress. (**a**) Relative root length. (**b**) Relative fresh weight. (**c**) Chlorophyll content. (**d**) Pn. (**e**) Gs. (**f**) Ci. (**g**) Tr.

In order to study the effects of salt stress on growth and Photosynthesis of transgenic Chrysanthemum, we measured the root length, fresh weight, chlorophyll content and leaf gas exchange parameters under salt stress. Salt stress inhibited the growth and development of chrysanthemum, WT and transgenic plants both showed root atrophy and fresh weight reduction, but the root length, fresh weight of transgenic plants decreased less, compared to WT (Fig. 5a,b). As shown in Fig. 5c, under different salt concentrations, the chlorophyll increased first and then decreased, and the transgenic plants decreased slightly than WT. With the increase of NaCl concentration, the net photosynthetic rate, stomatal conductance and transpiration rate of WT and transgenic plants both decreased, and the reduction range of transgenic plants was less than WT (Fig. 5d,e,g). On the contrary, intercellular CO_2_ concentration increased with salinity under salt stress, and the amplitude of WT was higher than that of transgenic plants (Fig. 5f). These results demonstrate that salt stress hindered the growth and photosynthesis of chrysanthemum, while the *DgWRKY5* transgenic plants had stronger salt tolerance compared with WT.

### Expression of abiotic stress-related genes in *DgWRKY5* transformed chrysanthemum plants

**Figure 6:**
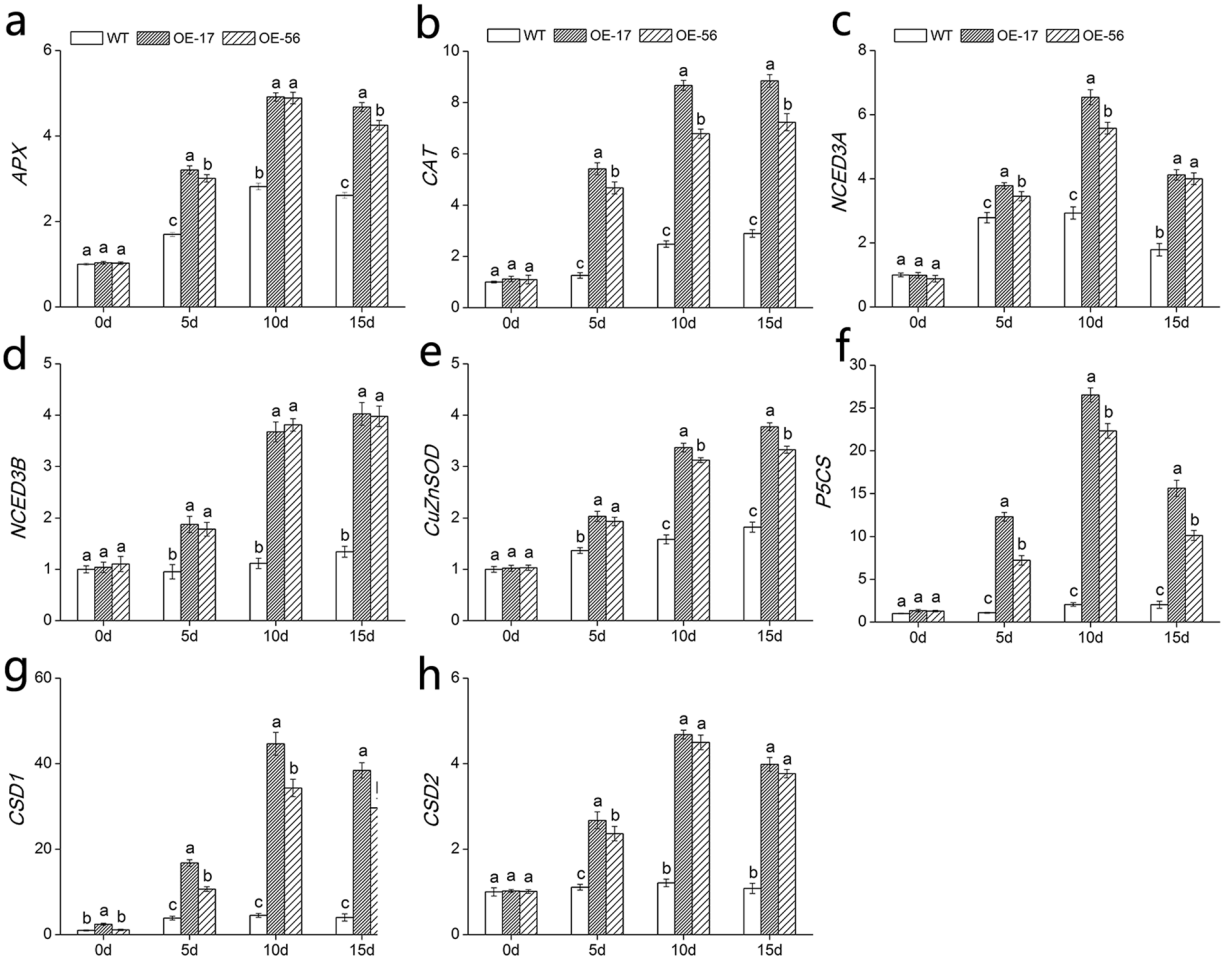
Expression of stress responsive genes in WT and *DgWRKY5* transgenic lines (OE-17 and OE-56) at various time points (0, 1, 5, 10 and 15d). EF1a was amplified as a control. Data represent means and standard errors of three replicates. Different letters above columns indicate (P < 0.05) differences between lines.

To reveal the underlying regulatory mechanisms of *DgWRKY5* transgenic lines in response to salinity stress, we investigated the relative expression level of eight stress-related genes through qRT-PCR, including *DgAPX*, *DgCAT*, *DgNCED3A*, *DgNCED3B*, *DgCuZnSOD*, *DgP5CS*, *DgCSD1* and *DgCSD2*. The expression levels of these genes were no obvious difference in wild type and transgenic lines during normal conditions (Fig. 6a–h). After salt stress, relative expression level of *DgAPX* and *DgCAT* genes which encoded ROS scavenging enzymes were significantly (*P < *0.05) up-regulated in *DgWRKY5* transgenic lines, comparing with WT plants increased by about two and three times, respectively (Fig. 6a,b). And the expression of ABA-responsive genes *DgNCED3A*, *DgNCED3B* and *DgCuZnSOD* had also greatly raised in transgenic lines compared to WT under salt treatment (Fig. 6c–e). Moreover, other abiotic stress-response genes, such as *DgP5CS*, which functions in osmotic adjustment, *DgCSD1*, and *DgCSD2* were all significantly (*P < *0.05) increased in transgenic plants than WT under salinity condition (Fig. 6f–h). Therefore, *DgWRKY5* may enhance salt tolerance of transgenic chrysanthemum by up-regulating expression levels of stress-related genes.

## Discussion

Previous studies have shown that WRKY transcription factors play an important role in regulating the response of plants to abiotic stress^15, 16^. In this research, we isolated and identified a transcription factor, *DgWRKY5*, from chrysanthemum. *DgWRKY5* contains two WRKY domains of WKKYGQK and two C2H2 zinc-fingers in sequence analysis (Fig. S2). The phylogenetic analysis showed that *DgWRKY5* is clustered with *AtWRKY26* and *AtWRKY25* from *Arabidopsis thaliana*, *TaWRKY2* from *Triticicum aestivum L*. and *OsWRKY30* from rice, all these genes belong to WRKY family group I (Fig. S3).

*DgWRKY5* was significantly induced by salt stress, which is similar with *AtWRKY25*, *AtWRKY33* in *Arabidopsis* thaliana and *TaWRKY2* in *Triticicum aestivum L* ^10, 17^. Moreover, the *DgWRKY5* transgenic chrysanthemum was more tolerant to salt stress which is similar to other WRKY transcription factors from same subgroup, including *AtWRKY26*, *AtWRKY25*, *OsWRKY30* and *TaWRKY2*, which also strengthen transgenic plants’ resistance to abiotic stress as described in the previous section. In addition, it is possible that the same subgroup transcription factors have analogous effects on abiotic stress. In addition, *DgWRKY5* is also strongly induced by multiple stresses, including ABA, H_2_O_2_ and GA. These results indicate that *DgWRKY5* is a novel transcription factor of WRKY family, which may participate in response to abiotic stress.

In response to salt stress, plant cells often tend to accumulate some organic molecules, such as proline, soluble sugar and soluble protein in the cytoplasm, to maintain a high osmotic pressure and ensure the plant can be still absorb moisture from the soil. The accumulation of proline is a protective measure taken by plants in order to fight against salt stress^18^. And it has been reported that plants with higher proline and soluble sugar had better resistant to stress^19^. In our research, we found that the contents of osmotic adjustment substances in transgenic plants were noticeably higher than WT plants. These data indicated that *DgWRKY5*-overexpression plants may enhance the ability to withstand salt stress in plants by increasing the content of osmotic adjustment substances.

High salinity leads to reactive oxygen species (ROS) accumulation, which may injure cytomembrane, cause mediate lipid peroxidation and more oxidative damage in plants^20^. It was reported that abiotic stress can causes lipid peroxidation, leading to accumulation of MDA^21^. The content of MDA can reflect the degree of harm brought about by salt stress on plants^22^. In this experiment, *DgWRKY5* transgenic plants produced a small amount of H_2_O_2_ and MDA in comparison with WT under salt stress. Protective substances include enzymes, such as SOD, POD and CAT. SOD is a primary O_2_^-^ scavenger, and CAT dismutates H_2_O_2_ into water and molecular oxygen, and POD decomposes H_2_O_2_ by oxidation of co-substrates such as phenolic compounds and/or antioxidants^23^. *DgWRKY5* transformed chrysanthemum plants contained highly activity of ROS scavenging enzymes (SOD, POD and CAT), compared with WT under non-stress condition and salt treatment. These results suggested that *DgWRKY5* could regulate the expression of ROS scavenging enzymes, enhance cell membrane protection system and weaken the membrane lipid peroxidation to improve the salt tolerance of plants.

Salt stress can cause plant growth and development to be seriously hindered, root length decreased and fresh weight reduction^24^. In our study, the higher the salinity was, the more inhibited the root length and fresh weight of chrysanthemum, but the inhibition of salinity was less on transgenic plants, compared with WT. Chlorophyll is the basic pigment for photosynthesis in plants, and its changes directly affect the photosynthesis of plants^25^. Salt stress increased chlorophyllase activity and promoted chlorophyll degradation, which caused chlorophyll content to decrease^26^. However, some studies have shown that salt stress can increase chlorophyll content significantly, it is considered that the binding between chlorophyll and chloroplast proteins in leaves is relaxed under salt stress, which makes chlorophyll easy to extract^27^. Our data revealed that *DgWRKY5* transgenic plants had higher chlorophyll content than WT, and the content was both increased under 100 mM NaCl stress. Plant photosynthesis is the basic process of material accumulation and physiological metabolism in plant production process^28^. It is also an important means to analyze environmental affecting plant growth and metabolism. In this study, the net photosynthetic rate (Pn), stomatal conductance (Gs) and transpiration rate (Tr) decreased, while intercellular CO_2_ concentration (Ci) increased under high salinity stress, it showed that the photosynthetic ability of mesophyll cells was further decreased, and the utilization of CO_2_ was lower, leading to CO_2_ excess and a corresponding decrease in photosynthetic product, so plants are more seriously damaged. However, these parameters of leaf gas exchange in *DgWRKY5* transgenic plants showed that it was more photosynthetic than WT and had stronger salt resistance. These results suggest that *DgWRKY5* transgenic plants may be resistant to salt stress by slowing salt damage to roots and enhancing leaf photosynthesis.

Under various stresses, transcription factors play important roles by modulating the target genes expression to strengthen the ability of plants stress tolerance^29^. *P5CS* gene, as a kind of osmotic protective agent, helps the plant to resist the change of osmotic imbalance under salt stress^30^. *CSD1* and *CSD2*, which are very effective on the detoxification of superoxide radicals^31, 32^. *NCED* is another important enzyme in the synthesis of ABA and has been determined to function in drought and salt stress^6^. In addition, *CuZnSOD* and *APX* have essential roles in enhancing the salt tolerance of sweet potato^33^. In this study, relative expression levels of stress responsive genes which were participated in oxidative stress-response (*DgCAT* and *DgAPX*), osmotic adjustment membrane protection (*DgP5CS*), and others mentioned above, were markedly up-regulated in *DgWRKY5* transgenic lines compared with wild-type under salt stress. The results suggested *DgWRKY5* may function as a constructive potential regulator of salt stress response pathway by controlling the expression of stress responsive genes.

In conclusion, *DgWRKY5*, a new WRKY transcription factor, was isolated from chrysanthemum and induced by abiotic stress. *DgWRKY5-*overexpression chrysanthemum showed enhanced salt tolerance compared with WT plants. In this study, we explored the physiological and molecular mechanism of *DgWRKY5*, and revealed that *DgWRKY5* played roles as a positive regulator in salt stress-response through regulating ROS scavenging and osmotic adjustment system as well as expression levels of stress-related genes.

## Methods

### Plant materials and stress treatments

The experimental plant material is wild-type chrysanthemum variety “Jinba” (*Dendronthema grandiform*). Chrysanthemum seedlings were grown in a greenhouse at 25 °C/16 h lights, 22°C/8 h dark cycle (light intensity of 200 μmol m-2s-1; relative humidity of 70%). For salinity and ABA treatments, chrysanthemum plants at the 6–7 leaves stage were used to culture in 200 mM NaCl or in 100 μM ABA media, respectively. For the H_2_O_2_ and GA treatments, the seedings were sprayed with 10 mM H_2_O_2_ or 5 μM GA, respectively. Untreated plants were used as controls. Samples of all the treatments were harvested at 0, 1, 3, 6, 12, and 24 h, frozen in liquid nitrogen promptly and stored at −80 °C for RNA extraction. For tissue-specific expression analyses, roots, stems and leaves of the same untreated seedlings were likewise collected. Each experiment contained three biological repeats.

### Cloning of *DgWRKY5* gene

On the basis of transcriptions data in chrysanthemum seedlings under non-stress conditions and salt stress conditions applying 454 high throughout sequencing technique, a large amount of salt-induced transcription was appraised. *DgWRKY5*, a novel transcription factor, was obviously brought about by salt stress. To obtain the total RNA sequence of *DgWRKY5*, leaves of seedlings at the 6–7 leaves stage were collected after 24 h under 400 mM NaCl treatment. Total RNA from chrysanthemum leaves was extracted with TRIzol reagent (Mylab, Beijing). The full-length cDNA of *DgWRKY5* sequence was obtained by PCR (polymerase chain reaction) and utilizing the gene specific primers (Table S1).

### Expression of *DgWRKY5* under salt treatment

We isolated total RNA from the chrysanthemum plants under salt stress treatment using TRIzol reagent (Mylab, Beijing) according to manufacturer’s protocol. Then RNA was used for first-strand cDNA synthesis with reverse transcriptase (Transgen, Beijing) according to the manufacturer’s protocol. Quantitative real-time PCR (qRT-PCR) was performed using SsoFast EvaGreen supermix (Bio-Rad, Hercules, CA, USA) and Bio-Rad CFX96TM detection system. The gene Elongation Factor 1α (*EF1α*) was used as a reference for quantitative expression analysis. A final 20 μL qPCR reaction mixture contained 10 uL SsoFast EvaGreen supermix, 2 uL diluted cDNA sample, and 300 nM primers. Then the reactions were incubated under the following program: 1 cycle of 95 °C for 30 s, 40 cycles of 15 s at 95 °C and 30 s at 60 °C, and a single melt cycle from 65 to 95 °C. Each reaction had repeated at least three times and negative controls without templates were detected in case of contamination. Relative expression levels were calculated by the 2^−ΔΔCT^ method^34^.

### Generation of *DgWRKY5* transgenic Chrysanthemum plants

In order to get the generation of *DgWRKY5* transgenic Chrysanthemum, *DgWRKY5* full-length cDNA was inserted into the expression vector pCAMBIA 2300 under the control of CaMV (cauliflower mosaic virus) 35 S promoter by replacing the gus gene. The obtained vector was transformed into chrysanthemum at leaf disk via Agrobacterium tumefaciens (strain LBA4404) according to An *et al*.^35^. The obtained *DgWRKY5* transgenic chrysanthemum lines (OE-17 and OE-56) were employed in subsequent experiments.

### Salt treatment of transgenic chrysanthemum

For the salinity tolerance experiment, three-week-old WT and *DgWRKY5* transgenic chrysanthemum seedlings (OE-17 and OE-56) were used. Chrysanthemum seedlings were watering with an increasing concentration of NaCl every fifth day over the following days: 100 mM on day 1–5, 200 mM on day 6–10, 400 mM on day 11–15, based on referring to the suggestion of Chen *et al*.^36^.Root length and fresh weight were measured at 0, 5, 10 and 15 days. The survival rate of the seedlings was calculated after 2 weeks recovery processes.

### Physiological changes in transgenic chrysanthemum

Leaves of the above WT and transgenic plants were harvested for biochemical analysis after 0 days, 5 days and 10 days of salt treatment. Accumulation of H_2_O_2_ was assayed as described by Alexieva *et al*.^37^. Content of MDA was mensurate following the method of Zhang *et al*.^38^. Accumulation of proline was estimated according to Liu *et al*.^39^. Accumulation of soluble protein and soluble sugar was measured following Wang *et al*.^40^. Activities of SOD and POD were measured following Pan *et al*.^41^. And CAT activities were assayed following Zhang *et al*.^42^. The chlorophyll content was determined following Qin *et al*.^43^. Leaf gas exchange parameters were measured following Khaled *et al*.^26^, setting the endogenous light intensity is 600μmol·m^−2^·S^−1^, the concentration of CO_2_ is 360 μL·L^−1^, the temperature is 25 °C.

### Expression of abiotic stress response genes in *DgWRKY5* transgenic chrysanthemum

In order to valuate the expression of abiotic stress-related genes, the RNA of WT and transgenic lines were extracted for reverse transcription to generate cDNA as described above. Then relative expression levels of several abiotic stress-related genes in *DgWRKY5* transgenic chrysanthemum were detected by qRT-PCR with *EF1α* served as the internal reference gene. Abiotic stress-response genes monitored were *DgAPX*, *DgCAT*, *DgNCED3A*, *DgNCED3B*, *DgCuZnSOD*, *DgP5CS*, *DgCSD1* and *DgCSD2*. All relevant primers in the article are listed in Table S1.

## Electronic supplementary material

Supplementary Information
