# Supplementary material for: Chrysanthemum WRKY gene DgWRKY5 enhances tolerance to salt stress in transgenic chrysanthemum
Source: Sci Rep. 2017 Jul 6;7:4799. doi: 10.1038/s41598-017-05170-x (PMC5500475; doi:10.1038/s41598-017-05170-x)
Supplement: Supplementary file 1 — Supplementary Information [file 41598_2017_5170_MOESM1_ESM.doc]

**Supplementary Information**

**Chrysanthemum WRKY gene *DgWRKY5* enhances tolerance to salt stress in transgenic chrysanthemum**

Qian-yu Liang, Yin-huan Wu, Ke Wang, Zhen-yu Bai, Qing-lin Liu*, Yuan-zhi Pan, Lei Zhang, Bei-bei Jiang

Department of Ornamental Horticulture, Sichuan Agricultural University, 211 Huimin Road, Wenjiang District, Chengdu, Sichuan 611130, P.R. China.

*Corresponding author; E-mail: qinglinliu@126.com;

Tel/Fax: +86-28-86290881.


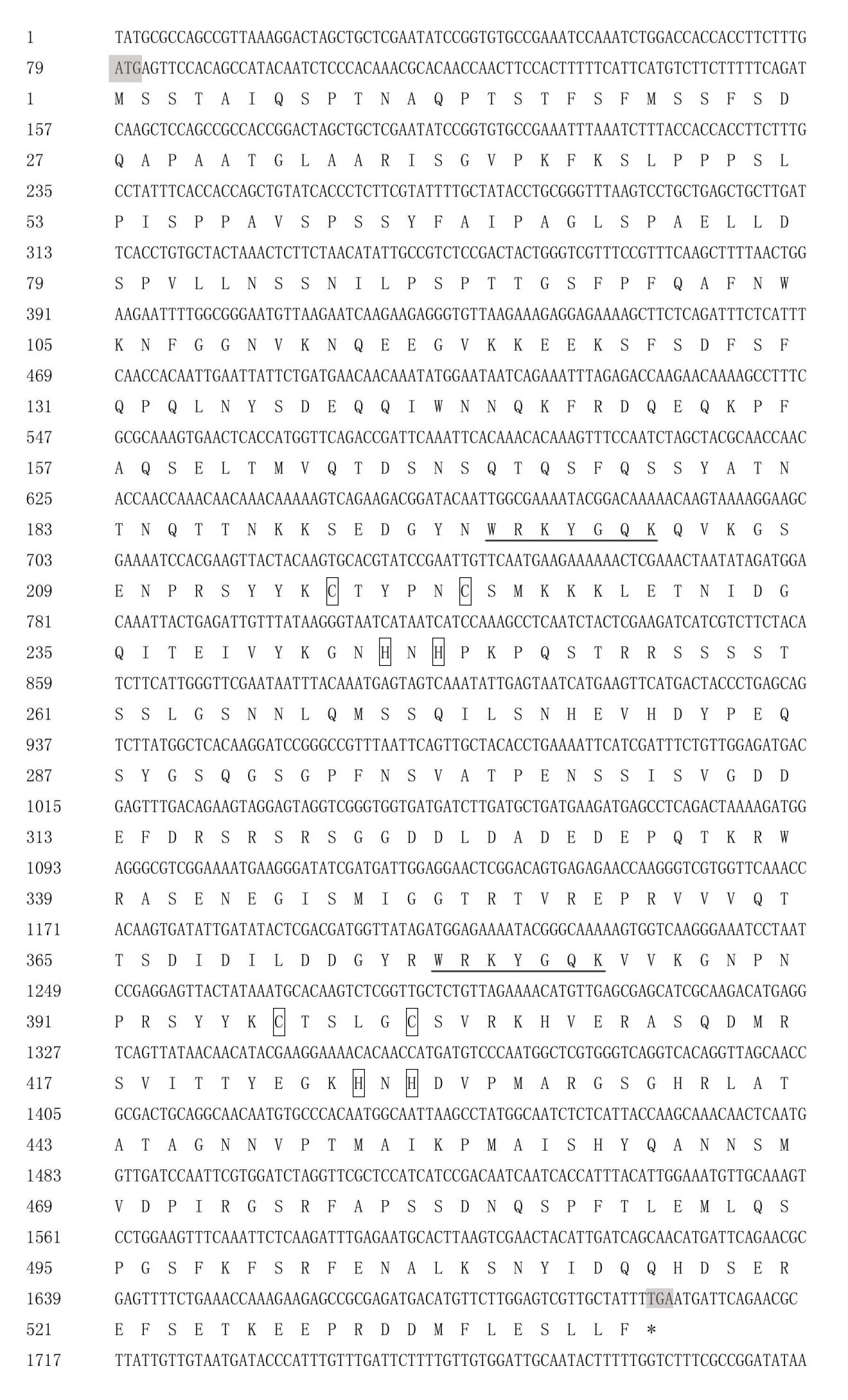


**Figure S1.** Nucleotide and deduced amino acid sequences of *DgWRKY5*. The WRKY domain was underlined, and the zinc finger motifs were boxed.


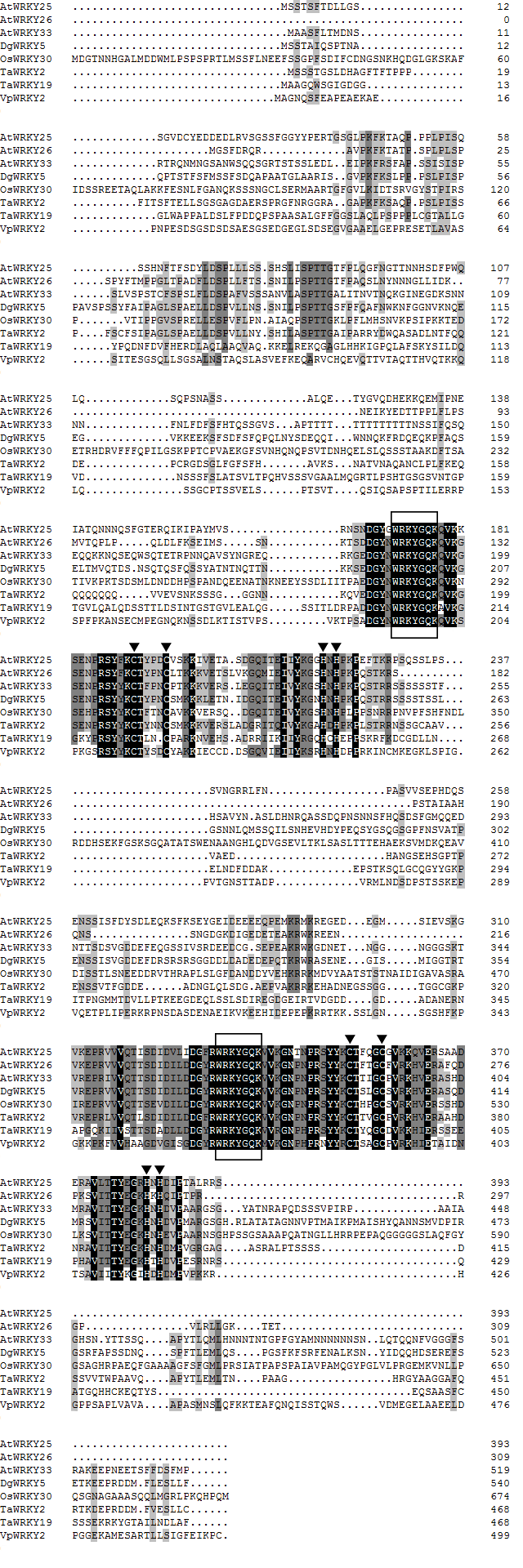


**Figure S2.** Alignment of the putative amino acid sequence of *DgWRKY5* with homologous proteins. The comparison was conducted by DNAMAN (version 6.0). Amino acid residues conserved in all sequences were shaded in black. The completely conserved WRKYGQK amino acids were boxed. The zinc finger motifs were indicated by arrowheads (▼). *Arabidopsis thaliana* (*AtWRKY25*, NP_180584; *AtWRKY26*, AAK28309; *AtWRKY33*, NP_181381); *Triticum aestivum* (*TaWRKY2*, EU665425；*TaWRKY19*, EU665430) ; *Oryza sativa* (*OsWRKY30*, NP_001062148); *Vitis pseudoreticulata* (*VpWRKY2*, GU565706)


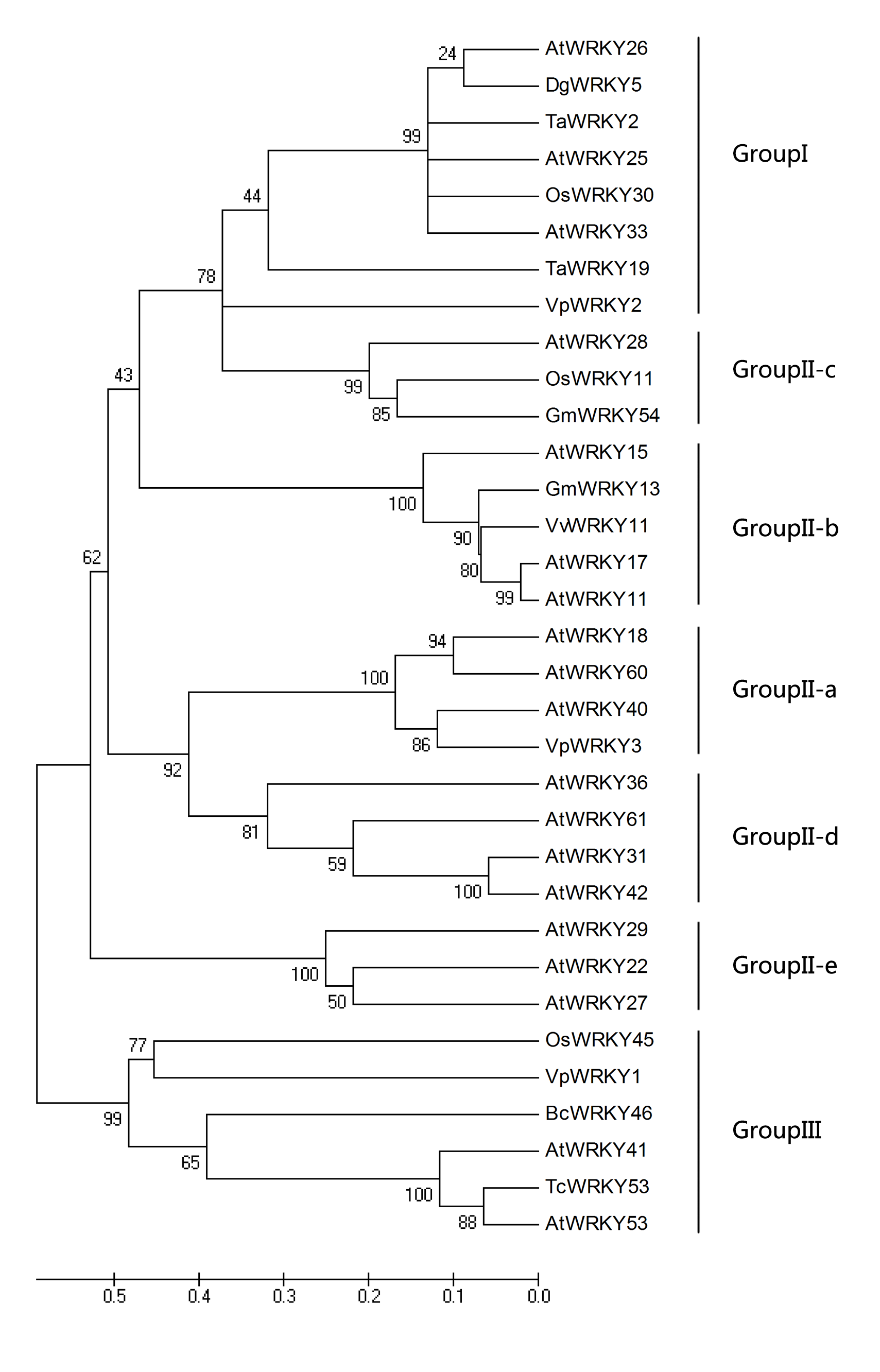


**Figure S3.** Phylogenetic analysis of relationships between *DgWRKY5* and WRKY proteins from other plant species. The amino acid sequences of the conserved WRKY domain region were subjected to the Bootstrap test of phylogeny by the MEGA program (ver 5). The accession numbers as follows: *VpWRKY1* (GQ884198), *VpWRKY2* (GU565706), *VpWRKY3* (JF500755) from *Vitis pseudoreticulata*; *VvWRKY11* (EC935078) from *Vitis vinifera*；*BcWRKY46* (HM585284) from *Brassica campestris;* *TcWRKY53* (EF053036) from *Thlaspi caerulescens*. *TaWRKY2* (EU665425), *TaWRKY19* (EU665430) from *Triticicum aestivum*; *GmWRKY13* (DQ322694), *GmWRKY54* (DQ322698) from *Glycine max*; *OsWRKY11* (AK108745), *OsWRKY30* (NP_001062148), *OsWRKY45* (AY870611) from *Oryza sativa*; *AtWRKY11* (NP_849559), *AtWRKY15* (NP_179913.1), *AtWRKY17* (NP_565574.1), *AtWRKY18* (NP_567882), *AtWRKY22* (AEE81999), *AtWRKY25* (NP_180584), *AtWRKY26* (AAK28309), *AtWRKY27* (NP_568777), *AtWRKY28* (NP_193551), *AtWRKY29* (AEE84774), *AtWRKY31* (NP_567644), *AtWRKY33* (NP_181381), *AtWRKY36* (NP_564976), *AtWRKY40* (NP_178199), *AtWRKY41* (NP_192845), *AtWRKY42* (NP_192354), *AtWRKY53* (NP_194112), *AtWRKY60* (NP_180072), *AtWRKY61* (NP_173320) from *Arabidopsis thaliana*.

**
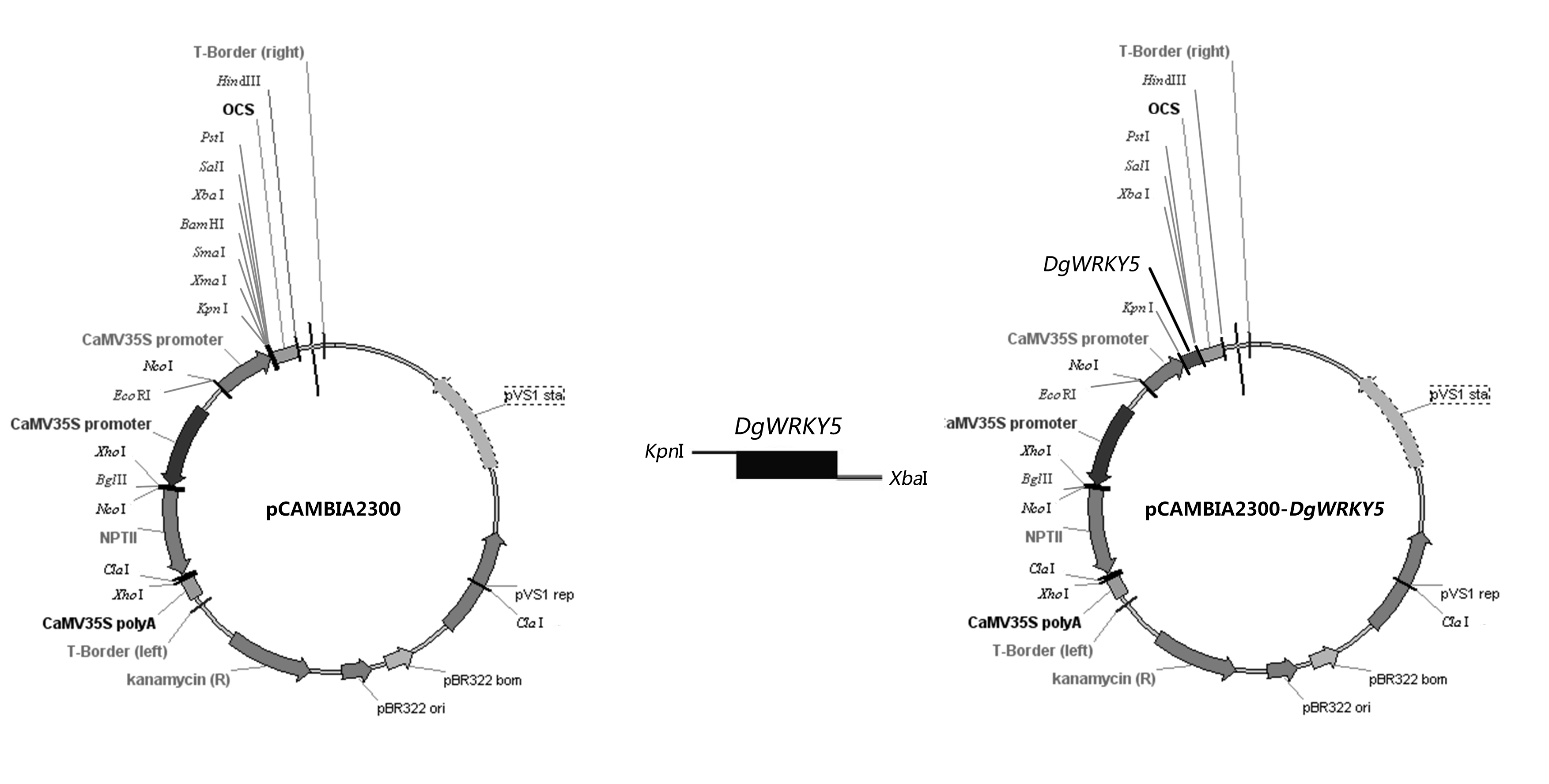
**

**Figure S4.** (**a**) Schematic diagram of the 35S::*DgWRKY5* fusion construct.


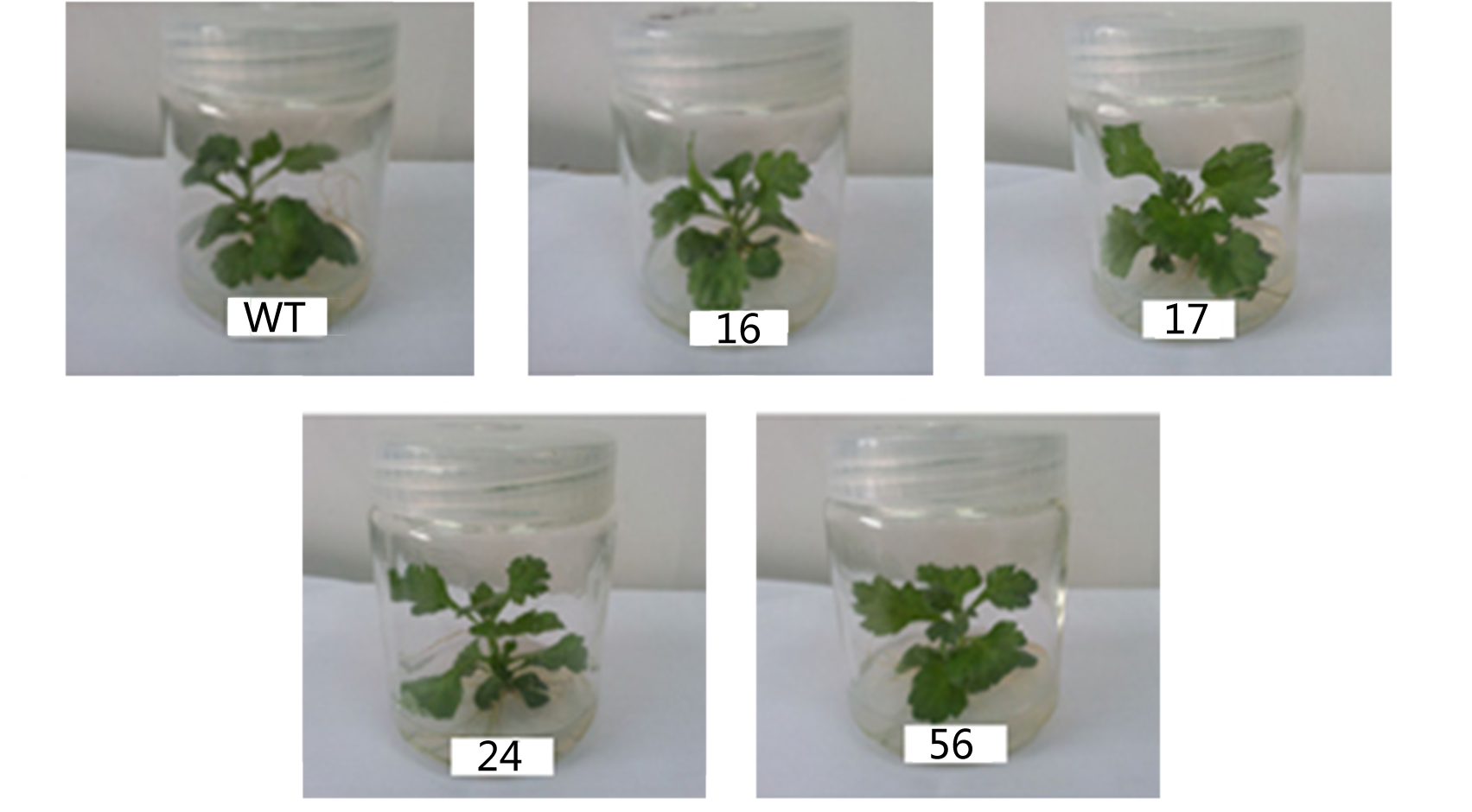


(**b**) *DgWRKY5* transgenic chrysanthemum lines regeneration.


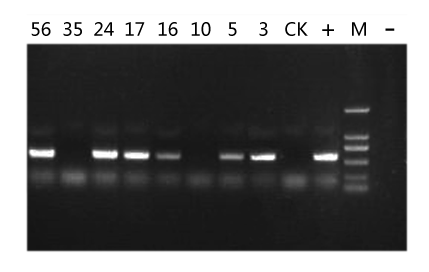


(**c**) PCR analysis of *DgWRKY5* transgenic chrysanthemum lines.

**Table S1.** **Primers used in this study.**

|  | **Forward Primers** | **Reverse Primers** |
| --- | --- | --- |
| **Primers Used for Cloning of *DgWRKY5*** | | |
| *DgWRKY5* | ATGAGTTCCACAGCCATACAATC | TTAAAATAGCAACGACTCCAAGAAC |
| **Primers Used to qRT-PCR** | | |
| *DgWRKY5* | GGAAGTTCATTTCATTTGGAGAGGA | CAAATATCATGCGATCATAGGCGTC |
| *EF1a* | TTTTGGTATCTGGTCCTGGAG | CCATTCAAGCGACAGACTCA |
| *DgCuZnSOD* | CCATTGTTGACAAGCAGATTCCACTCA | ATCATCAGGATCAGCATGGACGACTAC |
| *DgCAT* | TACAAGCAACGCCCTTCAA | GACCTCTGTTCCCAACAGTCA |
| *DgAPX* | GTTGGCTGGTGTTGTTGCT | GATGGTCGTTTCCCTTAGTTG |
| *DgP5CS* | TTGGAGCAGAGGTTGGAAT | GCAGGTCTTTGTGGGTGTAG |
| *DgNCED3A* | AGTATGGTGGTGAGCCGTTGTATCTAC | GCATTCACAATCTGGAGTTCGGACTTC |
| *DgNCED3B* | CATACTTGGCGATTGCGGAACCAT | GGCTCACCACCATACCTCTCATCAC |
| *DgCSD1* | TTCGTCCATCAGTCTAGTATCAAG | ATCACCACCACCACCACCTC |
| *DgCSD2* | AGTGAAGATGGACGAAAAAAGG | CTAGCAAAATGACCAACCCG |
